# Medical Segment Anything Model (MedSAM): methodological innovations, evaluation practices, and clinical applications

**DOI:** 10.1186/s12880-026-02600-z

**Published:** 2026-07-29

**Authors:** Wenjie Zhang, Liming Luo, Mengnan He, Molly Beestrum, Jiancheng Ye

**Affiliations:** 1 Weill Cornell Medicine, Cornell University, New York, NY USA; 2 Feinberg School of Medicine, Northwestern University, Chicago, IL USA; 3 Galter Health Sciences Library and Learning Center, Northwestern University, Chicago, IL USA

**Keywords:** Medical image segmentation, Foundation models, MedSAM, Promptable segmentation, Deep learning, Clinical AI, Scoping review, Fairness in medical AI

## Abstract

**Background:**

The Medical Segment Anything Model (MedSAM) has emerged as a prominent foundation model for medical image segmentation, prompting rapid growth in related research. Despite this proliferation, the literature remains fragmented across multimodal paradigms and lacks rigorous synthesis of methodological innovations, clinical safety evidence, algorithmic bias, and real-world implementation barriers—gaps that limit the field’s ability to translate algorithmic progress into clinical impact.

**Objective:**

To systematically identify, classify, and synthesize published research on MedSAM and its derivatives, providing a comprehensive overview of methodological developments, evaluation findings, and clinical translation progress.

**Methods:**

This scoping review followed the PRISMA-ScR guideline. We conducted a systematic search across six major databases (PubMed, Embase, Web of Science, Scopus, IEEE Xplore, ACM Digital Library) through June 2025.

**Results:**

From 1,482 initial records, 45 studies met inclusion criteria. Innovation-oriented studies (*n* = 32) were classified along four methodological axes spanning multimodal paradigms: Architecture Enhancement (encoder/decoder refinements for efficiency and precision), Prompt Strategy (textual, spatial, and multimodal prompting mechanisms), Efficiency and Deployability (quantization, distillation, and system integration), and Pipeline and Application (integration into clinical workflows). Evaluation-oriented studies (*n* = 13) revealed that MedSAM outperforms general-purpose SAM by 5–20% on structured modalities (CT/MRI) but lags behind task-specific networks for delicate structures. Notably, fairness analyses from two studies (combined *n* = 1,757 subjects) report Dice disparities of up to 10% across sex, age, and BMI strata, raising concerns about potential demographic bias that warrants further investigation in larger, multi-center cohorts before firm clinical-safety conclusions can be drawn. Real-world deployment is further constrained by prompt sensitivity (5–15% Dice variation), cross-modality domain shift, regulatory compliance gaps (FDA/CE marking, HIPAA, GDPR), and absent multi-center prospective validation, all of which represent principal implementation hurdles for clinical adoption.

**Conclusions:**

MedSAM represents a significant advancement in medical image segmentation, demonstrating a clear trajectory from large-scale architectures toward lightweight, prompt-aware, and deployable models. This synthesis provides a structured critical appraisal indicating that the evidence base remains at the proof-of-concept stage, with most studies retrospective and single-center, and emerging signals of algorithmic bias, unresolved cross-modality failure modes, and incomplete regulatory pathways represent safety and implementation considerations that should be addressed before multimodal MedSAM-based systems are more broadly deployed in clinical imaging workflows.

**Supplementary Information:**

The online version contains supplementary material available at https://doi.org/10.1186/s12880-026-02600-z.

## Introduction

Medical image segmentation represents one of the most critical tasks in computer vision and clinical applications, serving essential roles in organ delineation, target detection, and clinical decision-making. Traditional approaches such as graph cut [1], active contour [2], and level set methods [3] have achieved notable success in extracting organ boundaries. However, these methods rely heavily on manual annotations and strong prior constraints, limiting their adaptability to the substantial variability and complexity across clinical scenarios.

With the advent of convolutional neural networks, architectures such as U-Net [4] and V-Net [5] have achieved remarkable progress across various medical imaging tasks, including brain tumor, liver, and cardiac segmentation. Despite their strong performance on specific tasks, these models require substantial quantities of labeled data and exhibit limited cross-modality generalization. To address these limitations, researchers have increasingly turned to foundation models—large-scale pre-trained models designed to learn generalizable representations across diverse domains. In computer vision, the Segment Anything Model (SAM) [6] has emerged as an important development, enabling promptable segmentation through large-scale training and interactive input modalities such as points and bounding boxes. However, direct application of SAM to medical imaging encounters substantial constraints due to fundamental differences in imaging modalities and data distributions. These challenges have motivated the development of domain-adapted extensions tailored specifically to medical scenarios.

Medical Segment Anything Model (MedSAM) [7] represents an adaptation of SAM specifically designed for medical imaging tasks. Through retraining or structural adaptation of SAM on multimodal medical datasets, MedSAM aims to achieve generalized segmentation of organs, lesions, and anatomical regions across diverse modalities. Since its introduction in 2023, MedSAM research has rapidly expanded along two primary directions. The first encompasses methodological innovation, focusing on improving the model through architectural enhancements, prompt-design strategies, and deployability optimization. The second involves model evaluation, which systematically compares MedSAM with other segmentation frameworks across various tasks and datasets to assess its generalization capabilities and performance characteristics. Despite this progress, early evaluations have also exposed specific, recurring failure modes that motivate systematic review: sensitivity to prompt placement and quality, such that small perturbations in bounding-box or point prompts can produce disproportionate drops in segmentation accuracy; cross-modality collapse, in which performance degrades sharply when the model trained or validated predominantly on CT/MRI is applied to ultrasound, optical coherence tomography, or histopathology without modality-specific fine-tuning; and demographic performance variation that has only recently begun to be characterized. In clinical workflows, these failure modes are not merely academic, as a missed or mis-delineated boundary downstream of an unstable prompt or an unrecognized modality shift can translate directly into radiology reporting errors, inefficient re-review cycles, or flawed downstream measurements (e.g., tumor burden or organ volume) that propagate into treatment planning. These translational stakes, rather than algorithmic performance metrics alone, are the central motivation for the present review.

However, despite the proliferation of studies, a comprehensive synthesis systematically examining both methodological innovations and evaluation efforts remains absent. Innovation-oriented papers introduce diverse strategies, including architectural refinements, prompt design mechanisms, and decoder enhancements, yet few studies have summarized or compared these developments within a unified framework [8]. Similarly, evaluation-oriented studies assess MedSAM across different datasets, imaging modalities, and segmentation tasks, but no review has integrated these findings to reveal consistent patterns of generalization or identify persistent limitations. Consequently, a global overview connecting these research streams remains critically needed.

This study aims to provide a comprehensive understanding of MedSAM’s methodological landscape and evaluation ecosystem, establishing groundwork for future advancements in medical foundation models. The main contributions of this review include: (1) constructing a taxonomy of MedSAM-related studies, encompassing innovations in four explicit axes—Architecture Enhancement (encoder/decoder design), Prompt Strategy (spatial, textual, and multimodal prompting), Efficiency and Deployability (compression, quantization, and runtime design), and Pipeline and Application (clinical workflow integration)—that, unlike generic narrative surveys of SAM-based medical segmentation, explicitly separates methodological innovation from independent benchmarking and ties each axis to quantitative outcomes in a structured summary table; (2) comparing and analyzing evaluation results across multiple public medical datasets, examining MedSAM’s performance trends and limitations relative to conventional and emerging segmentation models; (3) identifying key challenges and outlining future research directions to advance medical foundation models.

## Methods

**Fig. 1:**
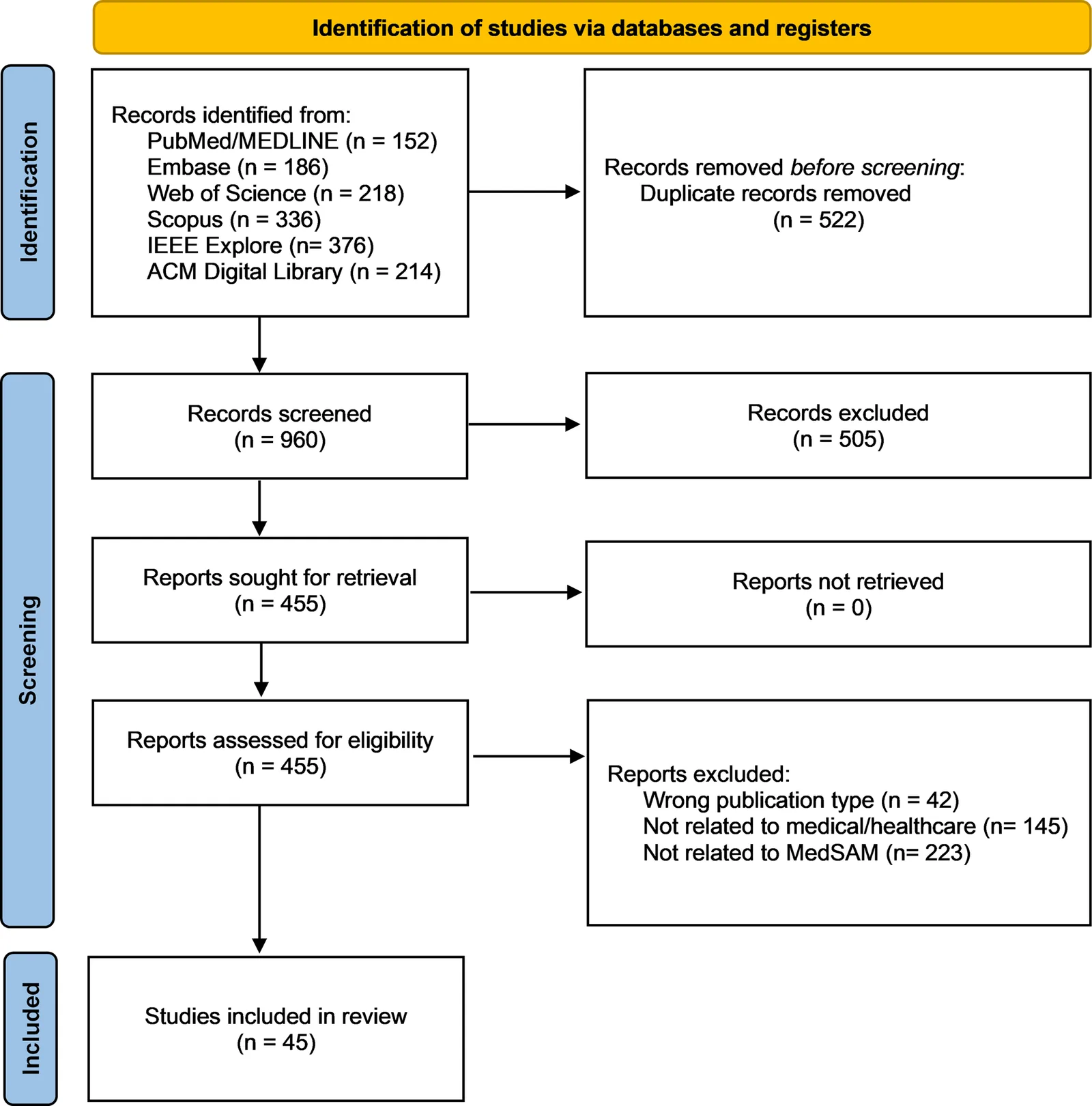
PRISMA flow diagram of the scoping review

This scoping review was conducted to systematically identify, classify, and analyze studies related to MedSAM. The review process followed the PRISMA extension for Scoping Reviews (PRISMA-ScR), as summarized in Fig. 1 [9]. A comprehensive literature search was performed across six major databases: PubMed/MEDLINE (*n* = 152), Embase (*n* = 186), Web of Science (*n* = 218), Scopus (*n* = 336), IEEE Xplore (*n* = 376), and the ACM Digital Library (*n* = 214). The search covered the period through June 2025, using combinations of controlled vocabulary and free-text terms such as “MedSAM” and “medical image segmentation”. Complete search strategies are provided in the Supplementary Table 1.

To ensure transparency and reproducibility, we established explicit inclusion and exclusion criteria. Eligible studies were required to be peer-reviewed journal articles or full-length conference papers focused on medical image segmentation that either used or modified MedSAM and were written in English with retrievable full text. Given the rapid pace of dissemination that characterizes this field, preprints (e.g., arXiv) were also eligible for inclusion provided they reported original, methodologically complete innovation- or evaluation-oriented results pertinent to MedSAM and satisfied the same full-text retrievability and minimum methodological-reporting requirements (i.e., a described dataset, model, and quantitative outcome) applied to peer-reviewed studies; conference proceedings papers formally peer-reviewed as part of edited volumes (e.g., the CVPR 2024 MedSAM on Laptop Challenge proceedings) were treated as peer-reviewed for inclusion purposes. Studies that could not be verified against these minimum reporting standards were excluded at full-text screening. Non-medical or general computer vision studies were excluded, as were posters, abstracts, patents, narrative reviews, and materials without retrievable full text. Screening was conducted by four researchers working in pairs, such that at least two independently reviewed each article; disagreements were resolved by discussion and adjudicated by a third reviewer when needed.

We further operationalized the review by dividing it into two primary research orientations. Innovation-oriented studies refer to those that modify the MedSAM framework or training strategy to produce novel models for medical segmentation. In contrast, evaluation-oriented studies do not alter the MedSAM architecture but systematically benchmark MedSAM across datasets, tasks, or imaging modalities to assess its generalization and stability. This orientation label was assigned according to each study’s stated primary objective rather than treated as a strict dichotomy: studies that introduced a structural, training, or prompting modification to MedSAM and reported the resulting model’s performance were classified as innovation-oriented even when they also benchmarked against baselines, because benchmarking in this context served to demonstrate the value of the proposed modification rather than to characterize MedSAM itself. Conversely, studies were classified as evaluation-oriented when MedSAM was used unmodified (zero-shot or with standard fine-tuning only) as one of several models under comparison. Two reviewers independently applied these criteria to each study, with disagreements adjudicated by a third reviewer; no study was assigned to both categories. The following sections present a taxonomy organizing innovation-oriented studies by methodological focus, alongside a comprehensive synthesis of evaluation-oriented studies highlighting benchmarking trends and comparative findings. Consistent with PRISMA-ScR guidance, a formal quality appraisal or risk-of-bias assessment of individual studies was not performed, because the primary purpose of the scoping review was to map the extent, range, and nature of available evidence rather than to pool effect estimates or grade evidence certainty.

## Results

In total, 1,482 records were initially retrieved. After removing 522 duplicates, 960 unique articles were screened based on titles and abstracts. During this stage, 505 records were excluded for failing to meet inclusion criteria. The remaining 455 reports underwent full-text assessment, with all texts successfully retrieved. Following eligibility evaluation, 410 studies were excluded, ultimately yielding 45 studies that satisfied all criteria for inclusion in this review.

### Taxonomy

**Fig. 2:**
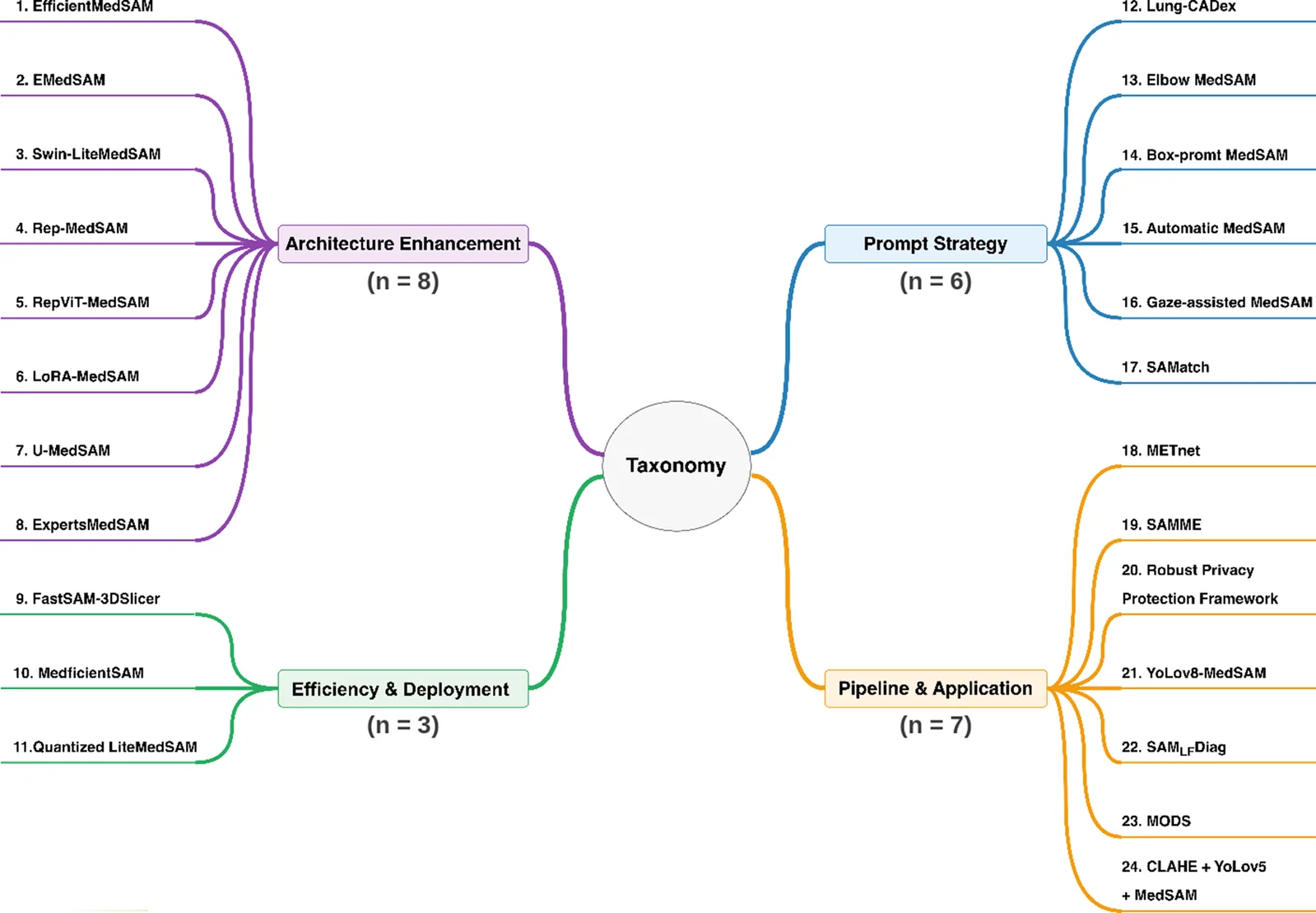
Taxonomy of innovation-oriented MedSAM studies. Each numbered prefix denotes the corresponding reference: 1. [10], 2. [11], 3. [12], 4. [13], 5. [14], 6. [15], 7. [16], 8. [17], 9. [18], 10. [19], 11. [20], 12. [21], 13. [22], 14. [23], 15. [24], 16. [25], 17. [26], 18. [27], 19. [28], 20. [29], 21. [30], 22. [31], 23. [32], 24. [33]

To systematically analyze innovation-oriented studies in MedSAM research, we developed a four-axis taxonomy comprising Architecture Enhancement, Prompt Strategy, Efficiency and Deployability, and Pipeline and Application (Fig. 2). This framework was designed to capture the primary directions along which MedSAM-related innovations have evolved and to provide a structured lens for cross-study comparison. The rationale for selecting these four axes was grounded in both theoretical and empirical considerations. From a theoretical perspective, they align with fundamental components of segmentation foundation models: network architecture, prompt mechanism, computational efficiency, and application workflow. Empirically, our review of representative MedSAM papers revealed that all methodological contributions could be categorized along one of these axes, indicating that the taxonomy comprehensively captures the methodological landscape. Operationally, two reviewers independently read the full text of each innovation-oriented study and assigned it to the axis corresponding to its single most prominent, headline methodological contribution (e.g., the modification the authors themselves emphasized in the title, abstract, and stated contribution); disagreements were resolved by discussion with a third reviewer. We recognize that several studies report contributions spanning more than one axis (for instance, a lightweight encoder evaluated together with a new prompting scheme); in such cases, each study was assigned to the axis representing its primary claimed novelty, while its secondary contribution is described narratively in the relevant subsection. Accordingly, each study appears only once in Table 1 under its primary axis.

**Table 1:** Summary of the included studies on MedSAM and its derivatives

Author	Year	Country/ Region	Dataset	Image Modality	Model	Baseline Model	MedSAM Role	Primary Performance	Key Implication
Chen [30]	2024	China	Ultralytics, Brain Tumor Dataset	MRI, CT	YOLOv8-MedSAM	-	Support	*Accuracy* 97.0%	Integrates YOLOv8 detection, MedSAM segmentation and CNN classification to boost tumor recognition without clicks
Cui [22]	2024	China	Elbow CT series	CT	Elbow MedSAM	MedSAM	Primary	*Median IoU* Humerus: 96.3% Ulna: 95.9% Radius: 95.0%	Automates MedSAM with bone-recognition prompts based on anatomical features
Dong [11]	2024	China	FLARE 2022, Lung CT, Breast X-ray, BraTS	CT, X-ray, MRI	EMedSAM	Unetr	Primary	*Dice Score* FLARE 2022: 88.9% vs. 84.7% Lung CT: 90.1% vs. 87.6% Breast X-ray: 89.1% vs. 87.4%BraTS: 89.5% vs. 87.2%	Speeds up MedSAM while maintaining SOTA accuracy through the lightweight encoder and med-adapter
Deshpande [34]	2024	US	BUSI, ISIC, CANDID	Ultrasound, Dermoscopy, X-ray	- (Pipeline/ Weak-Labeling with MedSAM)	UNet++	Primary	*Dice Score* BUSI: 46.6% vs. 38.9% ISIC: 84.8% CANDID: 91.0%	Feeds MedSAM with prompts generated from a few-shot-trained lightweight segmentation model
Gaillochet [24]	2024	Canada	HC, CAMUS, ACDC	MRI, Ultrasound	Automatic MedSAM	U-Net	Primary	*Dice Score* HC: 85.2% vs. 61.8% CAMUS: 88.4% vs.79.1% ACDC: 80.4% vs. 60.0%	Adds a lightweight prompt encoder that automatically generates prompt embeddings from the image itself
Wang [16]	2025	US	CVPR24 MedSAM on Laptop challenge	Multiple (11 modalities)	U-MedSAM	LiteMedSAM	Primary	*Dice Score* 86.1% vs. 82.6%	Replaces MedSAM’s ViT with EfficientViT and introduces an uncertainty-weighted loss to improve segmentation across diverse datasets
Zeng [35]	2024	China	FLARE 2022	CT	LoRA-Fine-tuned MedSAM	MedSAM	Primary	*Dice Score* 92.1% vs. 90.2%	Uses LoRA fine-tuning plus feature-selection and hybrid prompts to increase accuracy
He [36]	2024	China	DENTEX	X-ray	- (Pipeline/ MedSAM-based Autoprompt + Segmentation)	SAM	Primary	*Accuracy* 86.7% vs. 54.2%	Fine-tunes SAM for coarse localization, then feeds the centroid of its position mask as a point prompt into MedSAM to obtain high-precision impacted-tooth segmentation under minimal supervision.
Asiimwe [10]	2024	US	Kvasir-SEG	Endoscopy	EfficientMedSAM	MedSAM	Primary	*mean Average Precision* 49.1% vs. 45.7%	Uses Neural Architecture Search (NAS) to find an efficient, non-attention-based encoder to replace MedSAM’s ViT backbone, then uses Knowledge Distillation (KD) to transfer knowledge from the original MedSAM to achieve faster inference.
Shen [18]	2024	US	TotalSegmentator test set	3D CT volumes	FastSAM-3DSlicer	SAM-Med3D	Support	*Inference time* 0.730 s/vol vs.1.76 s/vol	Combines MedSAM and other SAM variants to a 3D slicer in real time segmentation
Pang [37]	2024	China	Mediastinum dataset	CT	- (Pipeline/ Bbox-guided MedSAM)	MedSAM	Primary	*Dice Score* 99.1% vs. 93.6%	Derives bounding-box prompts by randomly perturbing the ground-truth masks and uses these boxes to guide MedSAM segmentation
Sun [27]	2024	China	NSCLC cohort	CT	METnet	-	Support	*Accuracy* 74.6%	Uses MedSAM to segment lung tissue on CT and integrates clinical variables to predict MET dysregulation
Xie [38]	2024	China	Cervical Disc MRI	MRI	MedSAM	-	Support	*Accuracy* 89.51%	Uses fine-tuned MedSAM to produce intervertebral-disc masks for the radiomics classifier
Liu [23]	2024	US	BraTS2018	MRI	Box-prompt MedSAM	MedSAM	Primary	*Dice Score* 65.3% vs. 68.7%	Combines SBPG and PGSR to convert point annotations into box prompts for MedSAM segmentation
Wei [13]	2024	China	CVPR24 MedSAM on Laptop challenge	Multiple (11 modalities)	Rep-MedSAM	MedSAM	Primary	*Dice Score* 85.9% vs. 83.2%	Replaces MedSAM’s ViT encoder with RepViT and distills knowledge from the original MedSAM, achieving faster inference without sacrificing accuracy.
Zhao [29]	2024	China	ISIC 2020, COVID-19 Radiography, Brain Tumor	Dermoscopic, X-ray, MRI	Robust Privacy Protection Framework	-	Support	*Message Reconstruction Accuracy* 100.0%	Combines MedSAM segmentation, CLAHE enhancement and blind watermarking to embed patient identity with high imperceptibility and robustness
Roy [31]	2024	US	BUSI	Ultrasound	SAM_LF_Diag	-	Support	*Accuracy* 86.0%	Extracts features from the frozen MedSAM encoder, selects them via LASSO, and trains an MLP classifier for benign–malignant breast tumor classification
Liu [28]	2024	US	CT-MR Brain, CTACardio, and CTA Abdomen	CT,MRI	SAMME	-	Support	*Average Inference Time* 0.008	Extends SAMM by integrating MedSAM and other SAM variants, adding prompt propagation and 3D bounding-box prompting
Zhou [39]	2024	China	BraTS 2023	MRI	MedSAM	MedSAM	Primary	*Dice Score* 88.9% vs. 72.3%	Uses object-detection-generated bounding boxes as prompts to fine-tune MedSAM, markedly boosting brain-tumor segmentation accuracy
Xu [26]	2024	US	ACDC, BUSI, MRLiver	MRI, Ultrasound	SAMatch	UniMatch	Primary	*Dice Score* ACDC: 89.4% vs. 88.1% BUSI: 96.2% vs. 95.6% MRLiver: 80.0% vs. 75.0%	Unifies SAM and MedSAM with Match-based semi-supervision to auto-generate prompts and refine pseudo-labels
Valensi [40]	2024	Israel	2,427 B-mode LUS scans from Tel Aviv Sourasky MC	Ultrasound	MedSAM	MedSAM zero-shot	Primary	*Dice Score* 77.0% vs. 67.0%	Combines tracker-aided semi-supervised detection with auto box prompts and foundation models
Le [19]	2024	Vietnam	CVPR24 MedSAM on Laptop challenge	Multiple (11 modalities)	MedficientSAM	LiteMedSAM	Primary	*Dice Score* 86.4% vs. 83.2%	Accelerates medical segmentation via lightweight architecture distillation and C++ inference optimization
Lu [20]	2024	China	CVPR24 MedSAM on Laptop challenge	Multiple (11 modalities)	Quantized LiteMedSAM	LiteMedSAM	Primary	*Dice Score* 83.5% vs. 83.3%	Accelerates medical segmentation on commodity hardware via quantization-aware training and deployment optimization
Gao [12]	2024	Netherlands	CVPR24 MedSAM on Laptop challenge	Multiple (11 modalities)	Swin-LiteMedSAM	LiteMedSAM	Primary	*Dice Score* 86.7% vs. 83.8%	Integrates multi-prompt strategies with a lightweight Swin Transformer for efficient medical segmentation.
Khaerdinova [25]	2025	Russia	WORD	CT	Gaze-assisted MedSAM	MedSAM	Primary	*Dice Score* 92.5% vs. 81.7%	Leverages eye gaze as an intuitive prompt for interactive segmentation correction.
Qasrawi [33]	2025	Palestine	Dunya Women’s Cancer Center Dataset (In-house)	Ultrasound	CLAHE + YOLOv5 + MedSAM	AMS-PAN	Primary	*Dice Score* 98.8% vs. 80.7%	Combines CLAHE-based enhancement with detector-driven box prompts for MedSAM and an edRVFL classifier, forming a practical end-to-end ultrasound workflow for early breast-cancer triage and decision support
Hu [15]	2025	UK	AMOS, BTCV, MultiTalent	CT, MRI	LoRA-MedSAM	MedSAM	Primary	*Dice Score* AMOS: 86.1% BTCV: 80.6% MultiTalent: 84.0%	Applies LoRA to enable parameter-efficient task adaptation of MedSAM, reducing fine-tuning resource requirements for faster customization under constrained compute
Shaukat [21]	2024	Pakistan	LIDC, LUNGx	CT	Lung-CADex	-	Support	*Accuracy* LIDC: 71.0% LUNGx: 70.6%	Replaces box and point prompts with prefix-tuned text prompts on a frozen MedSAM to enable downstream tumor diagnosis
Ali [14]	2024	Canada	CVPR24 MedSAM on Laptop challenge	Multiple (11 modalities)	RepViT-MedSAM	MedSAM	Primary	*Dice Score* *85.3%*	Uses RepViT encoder to enable CPU-friendly inference, while distillation and fine-tuning improve accuracy
Zhi [17]	2025	China	CVPR24 MedSAM on Laptop challenge	Multiple (11 modalities)	ExpertsMedSAM	LiteMedSAM	Primary	*Dice Score* 58.4%	Improves funduscopic segmentation under Scribble-Prompt while retaining cross-modality stability.
Nazzal [41]	2024	Spain	MSD, KiTS, COVID-19	CT	MedSAM (Test-Time Augmentation)	MedSAM, DeepLabv3+	Primary	*Dice Score* MSD: 93.6% KiTS: 93.8% COVID-19–20: 74.5%	Introduces an inference-time augmentation and fusion scheme to bolster MedSAM’s robustness without any retraining
Swinburne [32]	2024	USA	Internal:PACS-derived lung cancer dataset, External: LIDC-IDRI	CT	MODS pipeline	SAM, MedSAM	Primary	*Dice Score* Internal: 82.2% External: 80.4%	Shows that clinical PACS-mined annotations and foundational segmentation models can build robust pipelines with minimal manual labeling

Note: “–” in the Model column indicates that the study did not assign a specific model name; “–” in the Baseline Model column indicates that no baseline comparison was reported in the original publication

Specifically, Architecture Enhancement encompasses structural modifications designed to improve representation learning and feature fusion. Prompt Strategy focuses on the design and integration of prompts, including points, bounding boxes, text, or multimodal cues that guide the segmentation process. Efficiency and Deployability captures efforts to improve computational feasibility through lightweight design, knowledge distillation, quantization, or real-time adaptation. Finally, Pipeline and Application reflects the embedding of MedSAM within broader medical workflows, such as quantitative analysis and clinical decision support systems. It should be noted that Fig. 2 illustrates the structural logic of MedSAM’s methodological innovations rather than providing an exhaustive catalog of all studies. Only works that explicitly proposed a named model or offered reproducible architectural descriptions are visualized in the figure. Several studies that fine-tuned or adapted MedSAM without assigning a distinct model name are not displayed in Fig. 2 to avoid ambiguity, though they are comprehensively summarized in Table 1 for completeness and transparency. To aid interpretation of the relative weight of each methodological direction, Fig. 2 reports the number of visualized studies within each axis.

Building upon this taxonomy, Table 1 provides a comprehensive summary of all innovation-oriented studies included in this review, detailing their datasets, modalities, methodological focuses, and performance metrics. Because the included studies report a variety of outcome metrics (e.g., Dice score, accuracy, mAP, inference time) that cannot be meaningfully converted to a single common metric without access to raw data, full quantitative standardization was not possible; we have instead standardized the presentation format itself so that, wherever a comparison is reported, the metric name is given first followed by the proposed model’s value versus the baseline’s value (e.g., “Dice Score 86.1% vs. 82.6%”) using consistent “vs.” notation throughout, to facilitate at-a-glance comparison across rows. Based on content analysis of innovation-oriented papers, these studies are categorized along the four methodological axes. Together, these axes collectively delineate the innovation landscape of MedSAM, spanning from input prompt design and model refinement to efficiency optimization and clinical integration. Each methodological axis is discussed in detail in the following subsections, highlighting representative innovations, their technical motivations, and performance outcomes.

**Fig. 3:**
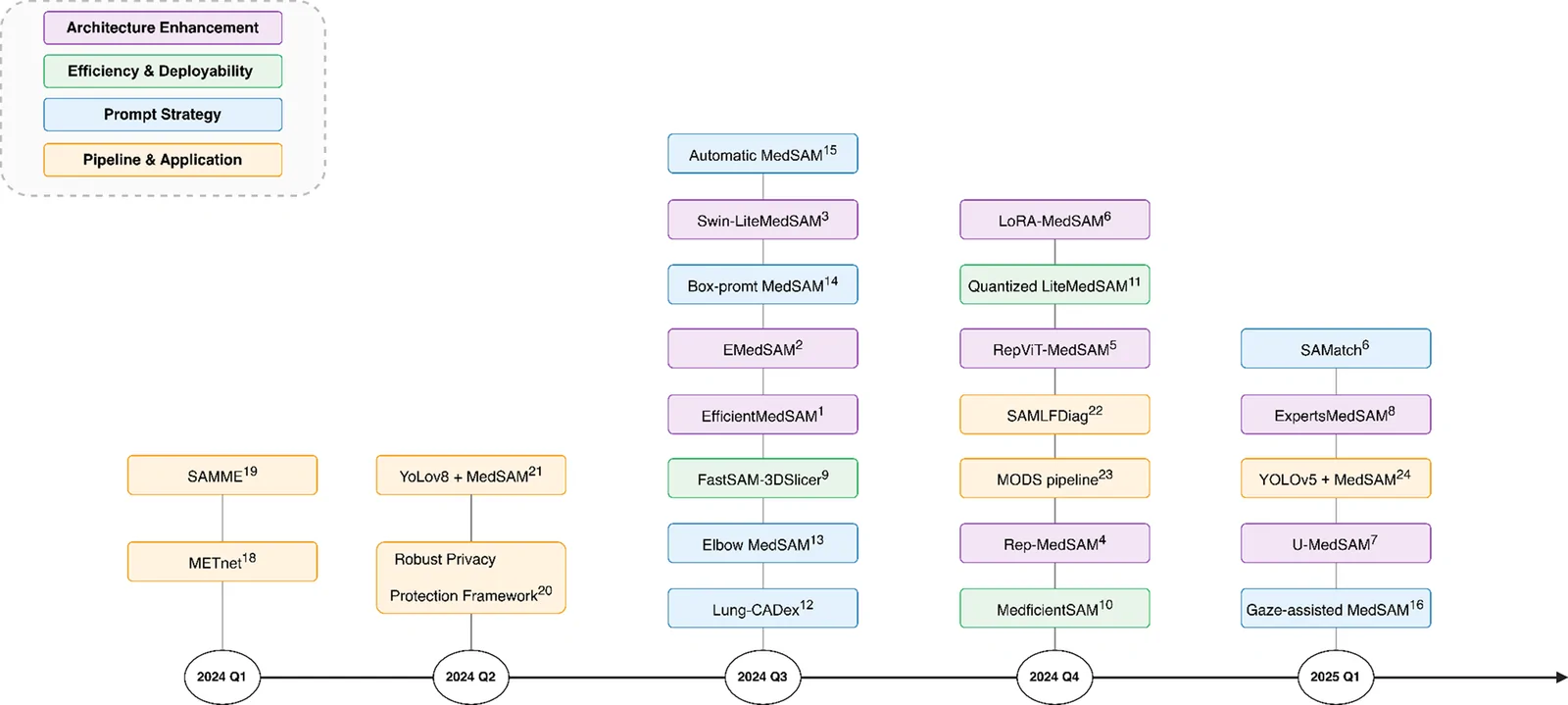
Timeline of MedSAM-related studies. Each study is color-coded according to its primary methodological focus. “Q” denotes the calendar quarter

In addition to the conceptual taxonomy, Fig. 3 presents a chronological timeline summarizing the evolution of MedSAM-related studies included in this review. This visualization contextualizes the methodological taxonomy by illustrating when each method emerged over time, highlighting the rapid diversification and maturation of MedSAM research.

### Architecture enhancement

Architectural enhancement efforts in MedSAM have primarily focused on refining both encoder and decoder components to balance segmentation accuracy, computational efficiency, and robustness.

On the encoder side, recent studies have aimed to improve representation learning while reducing computational cost through lightweight, reparameterized backbones. EfficientMedSAM [10] and EMedSAM [11] replace the original ViT-H encoder with TinyViT variants combined with teacher-student distillation, achieving comparable Dice scores while reducing inference latency. RepViT-MedSAM [14] extends this concept by introducing structural reparameterization, further accelerating runtime without compromising segmentation fidelity. In parallel, LoRA-MedSAM [15] explores parameter-efficient fine-tuning by incorporating Low-Rank Adaptation modules into MedSAM’s image and prompt encoders, reducing trainable parameters by approximately 87.5% and shortening fine-tuning time across multiple datasets while modestly improving Dice performance. These advances demonstrate that efficiency in MedSAM can be achieved not only through architectural simplification but also through optimization-level compression, enabling faster and more adaptable domain transfer under limited computational resources.

While encoder modifications emphasize efficiency and adaptability, decoder refinements primarily aim to enhance spatial precision, boundary consistency, and cross-modality robustness. Several works modify the decoder to better fuse hierarchical context and capture fine-grained anatomical structures. Swin-LiteMedSAM [12] adopts a hierarchical skip-connection decoder that integrates multi-scale attention, improving Dice and boundary accuracy in the CVPR 2024 MedSAM on Laptop Challenge [42]. U-MedSAM [16] introduces uncertainty-aware optimization within the decoding process, employing Sharpness-Aware Minimization to stabilize training across noisy or heterogeneous modalities. ExpertsMedSAM [17] further enhances adaptability through a modular decoder design, where expert branches specialize for different organs or imaging modalities. Although these methods improve segmentation stability and cross-domain generalization, they also increase model complexity and tuning costs, indicating that enhanced robustness sometimes comes at the expense of architectural simplicity and maintainability.

Overall, the evolution of MedSAM architecture reflects a dual trajectory: encoder enhancement studies prioritize compactness and adaptability, while decoder enhancement innovations advance spatial detail and robustness. Together, these developments mark a transition from computationally intensive foundation models toward lightweight, precision-driven medical segmentation frameworks.

### Prompt strategy

Building upon SAM’s promptable segmentation paradigm, MedSAM and its derivatives have extended the prompting mechanism to incorporate multimodal signals and automatic interactions. The overarching goal of these studies is to enhance adaptability and usability by transitioning from manually guided prompts to more intelligent, multimodal, and autonomous prompting schemes aligned with clinical workflows.

From an input modality perspective, prompt strategies in MedSAM can be grouped into textual, spatial, and multimodal categories. Text-based prompting leverages semantic or linguistic knowledge to guide segmentation. Lung-CADex [21] replaces traditional visual prompts with Contrastive Language–Image Pre-training (CLIP)-based [43] textual embeddings via prefix tuning, enabling fully automatic, zero-shot segmentation of lung nodules. By aligning textual prompts such as “lung nodule” with radiomic features, it achieved a sensitivity of 0.86, surpassing several fully supervised baselines on the Lung Image Database Consortium (LIDC) [44] and LUNGx Challenge (LUNGx) [45] datasets. Spatial prompting remains widely adopted due to its simplicity and interpretability. Elbow MedSAM [22] demonstrates that anatomically constrained bounding-box prompts can deliver expert-level accuracy in bone segmentation, while Box-Prompted MedSAM [23] introduces a semantic box-point refinement loop using a Semantic Box-Prompt Generator (SBPG) and Prompt-Guided Spatial Refinement (PGSR) module, enabling point-supervised learning to approach box-level precision. Hybrid multimodal prompting further combines textual and spatial cues, improving alignment between semantic intent and anatomical localization.

Regarding automation and interaction, prompt strategies have evolved from manual to semi-automatic and fully automatic paradigms [46]. Gaze-Assisted MedSAM [25] exemplifies natural, hands-free interaction by translating real-time gaze coordinates into implicit point prompts. Fine-tuned on abdominal CT data, it improved segmentation accuracy for complex cases by nearly 62% compared with traditional box interaction, while significantly reducing correction time. Automatic MedSAM [24] generates bounding boxes or point prompts from pre-segmented masks, eliminating human input and enabling autonomous region-of-interest detection. These methods collectively reduce the cognitive and operational burden on clinicians, moving MedSAM closer to seamless integration into clinical imaging pipelines.

Across studies, prompt strategy development in the MedSAM ecosystem demonstrates trade-offs among precision, automation, and usability. Spatial prompts such as bounding boxes and points yield the most accurate boundary delineation but require substantial manual interaction. Text-based prompts achieve superior cross-modality generalization and require minimal user effort, but their effectiveness depends on well-aligned semantic embeddings and comprehensive medical vocabularies. Automatic and multimodal prompting frameworks offer the most promising compromise, combining semantic understanding with contextual localization to deliver near-manual accuracy while substantially reducing annotation time. These hybrid approaches represent a transition toward human-in-the-loop autonomy, where clinician input is progressively reduced without sacrificing interpretability or control.

### Efficiency and deployability

Research on efficiency and deployability in the MedSAM landscape has increasingly focused on method-driven optimization to enable practical, resource-aware segmentation. These studies pursue deployability by improving computational throughput, memory efficiency, and inference stability through three complementary dimensions: system-level integration, architectural refinement, and precision-aware optimization.

At the system level, integration-oriented designs such as FastSAM-3DSlicer [18] embed MedSAM into established medical imaging ecosystems. By coupling uncertainty quantification with interactive 3D visualization within the 3D Slicer platform, clinicians can review, edit, and propagate volumetric masks in real time. This demonstrates that a model’s deployability depends not only on compression or computational efficiency but also on workflow-aware design that enhances usability, interpretability, and clinical trust. At the architectural level, models such as MedficientSAM [19] re-engineer MedSAM’s heavy ViT-B encoder via knowledge distillation into a lightweight EfficientViT backbone, while reimplementing the inference pipeline in C++ with OpenVINO acceleration. This dual optimization achieves over 5× speed-up and substantial memory reduction, illustrating how software redesign can deliver real-time inference without sacrificing segmentation fidelity. At the parameter level, Quantized LiteMedSAM [20] adopts quantization-aware training (QAT) to compress parameters to 8-bit precision while preserving numerical stability and cross-modality consistency. Compared to post-training quantization, QAT maintains the original model’s Dice coefficient and Normalized Surface Dice (NSD) performance while reducing power consumption and memory load, enabling stable CPU-based inference within an 8 GB limit. This highlights that energy efficiency and reproducibility can emerge from algorithmic precision control.

Table 2 summarizes the reported inference time, memory usage, and Dice performance of efficiency-oriented MedSAM variants. The inference time for FastSAM-3DSlicer was not reported numerically, as the authors described only real-time CPU performance within the 3D Slicer interface.

**Table 2:** Summary of efficiency-oriented MedSAM variants

Model	Inference Time on CPU	Memory Usage	Dice Performance
FastSAM-3DSlicer	Not numerically reported	8GB	0.84
MedficientSAM	0.38 s/slice	8–10 GB	0.86
Quantized LiteMedSAM	0.50 s/slice	8GB	0.84

Note: Memory usage and quantitative Dice performance for FastSAM-3DSlicer was not reported

Overall, recent efficiency-oriented studies demonstrate that MedSAM’s deployability depends on methodological combination across architecture, training, and runtime design. The integration of knowledge distillation, quantization-aware optimization, and runtime refactoring shows that computational feasibility can be achieved without compromising segmentation fidelity. These approaches collectively shift focus from pure model compression toward a more holistic notion of deployability that balances accuracy, latency, and usability within realistic clinical constraints. Rather than treating efficiency as a separate engineering goal, recent works increasingly regard it as an intrinsic part of model design, aligning algorithmic scalability with clinical practicality. These findings suggest that deployability depends on joint optimization of model design, runtime performance, and user interaction.

### Pipeline and applications

The Pipeline and Application component highlights how MedSAM is being integrated into broader medical imaging workflows, transforming it from an isolated segmentation tool into a modular component of diagnostic and decision-support pipelines. Across different modalities and tasks, recent studies consistently demonstrate MedSAM’s versatility in integrating with detection, classification, and data management systems.

Diagnostic-oriented pipelines such as METnet [27] exemplify this integration, where MedSAM-derived lesion features are used to predict genetic dysregulation in lung cancer non-invasively. Similarly, SAMME [28] embeds SAM and MedSAM into 3D Slicer, providing real-time volumetric segmentation and customizable fine-tuning interfaces, effectively bridging research models and radiology practice. These works illustrate MedSAM’s potential adaptability and the feasibility of coupling segmentation with radiogenomic or clinical visualization modules. In detection-guided and multimodal pipelines, MedSAM has been increasingly adopted as a segmentation backbone that interfaces with detection, classification, and enhancement modules. YOLOv8-MedSAM [30] integrates promptable segmentation with YOLO-based lesion detection and CNN-driven classification, achieving 97% diagnostic accuracy on photoacoustic brain tumor imaging. Similarly, CLAHE + YOLOv5 + MedSAM [33] extends this paradigm to ultrasound analysis, using contrast-limited adaptive histogram equalization (CLAHE) for image enhancement and hybrid spatial and text prompts for improved lesion delineation, reaching a Dice score of 0.99 and classification accuracy above 0.90 on 850 clinical cases.

Beyond detection-assisted workflows, SAM_LF_Diag [31] repurposes MedSAM’s encoder embeddings for breast ultrasound classification, outperforming conventional CNNs and demonstrating the potential of segmentation encoders as discriminative medical feature extractors. The Robust Privacy Protection Framework [29] integrates MedSAM for lesion localization before applying contrast enhancement and blind watermark embedding, thereby preserving diagnostic integrity while concealing patient identity. At a larger operational scale, MODS [32] represents an end-to-end workflow that connects hospital-level Picture Archiving and Communication System (PACS) data mining, YOLO-based lesion detection, and MedSAM-driven segmentation into a self-improving loop for automated labeling and model retraining. This design exemplifies how MedSAM can evolve from a standalone model into a dynamic component of continuous clinical data pipelines.

Despite these promising applications, a critical limitation shared by most studies is the lack of clinical-level validation. The majority rely on retrospective or single-institution datasets, often under controlled imaging conditions with limited modality diversity and no longitudinal or multi-center evaluation. Although these results confirm the algorithmic feasibility and flexibility of MedSAM-based systems, they provide limited evidence regarding real-world reliability, workflow efficiency, and safety of human-AI interaction. To date, only a few systems, such as SAMME and MODS, have demonstrated integration into clinical software environments, but even these remain at pilot or simulation stages rather than full clinical deployment. Bridging this gap between technical innovation and clinical validation will require prospective, clinician-in-the-loop studies to evaluate generalization, usability, and patient safety before MedSAM-based pipelines can be reliably adopted in daily medical practice.

### Dataset

To support reproducibility, benchmarking, and future dataset selection, Table 3 provides a comprehensive summary of all datasets used in the reviewed MedSAM-related studies. The table focuses exclusively on open-access or registered public datasets, while private, institution-specific, or non-disclosed clinical datasets are excluded to maintain transparency and reproducibility. It serves as a reference resource for the research community, mapping the landscape of imaging modalities, organ systems, and data accessibility involved in MedSAM development and evaluation, allowing future researchers to quickly identify suitable datasets for replication, cross-domain validation, and generalization testing.

**Table 3:** Summary of public datasets used in MedSAM-related studies

Organ Name	Dataset	Modality	Focus	Accessibility
Brain	Multimodal Brain Tumor Segmentation Challenge (BraTS series) [47]	MRI	Glioma	Public registered (test labels withheld)
	White Matter Hyperintensity Segmentation Challenge (WMH) [48]	MRI	White-matter hyperintensities	Public registered (test labels withheld)
	Ischemic Stroke Lesion Segmentation Challenge (ISLES series) [49]	MRI	Acute and sub-acute ischemic stroke lesion segmentation	Public registered (test labels withheld)
	Multiple Sclerosis Segmentation Challenge (MSSEG series) [50] 3D Magnetic Resonance Multiple Sclerosis dataset (3D-MR-MS) [51]	MRI	Multiple Sclerosis (MS) lesions	Public by request (test labels withheld)
Eye	Optical Coherence Tomography Image Dataset Based on Macular Hole Manual Segmentation (OIMHS) [52]	OCT	Macular hole and retinal layer	Public
	Annotated Retinal OCT Images Database for Joint Retinal-Layer and Fluid Segmentation (AROI) [53]	OCT	Retinal layers and intra-/subretinal fluid	Public by request
	Digital Retinal Images for Vessel Extraction Dataset (DRIVE) [54]	Fundus photography	Retinal vessel	Public
Teeth	Dental Enumeration and Diagnosis on Panoramic X-rays Challenge (DENTEX) [55]	X-ray	Tooth instance segmentation and dental abnormalities	Public registered
Chest	Imaging Dataset for Type-B Aortic Dissection (ImageTBAD) [56]	CTA	Type-B aortic dissection	Public
	Japanese Society of Radiological Technology Chest Radiograph Database (JSRT) [57]	X-ray	Lung nodule	Public registered
	COVID-19 Radiography Database [58]	X-ray	COVID-19 vs. normal/other-pneumonia classification	Public
	Chest Abnormality Nodule Detection Dataset (CANDID) [59]	X-ray	Pneumothorax, acute rib fracture, and intercostal chest tube	Public
Lung	Lung Image Database Consortium and Image Database Resource Initiative (LIDC-IDRI) [60] LUNGx Challenge Dataset [45]	CT	Pulmonary nodule	Public registered
Heart	Automatic Cardiac Diagnosis Challenge (ACDC) [61]	MRI	LV cavity, RV cavity, and myocardium segmentation at ED/ES	Public registered (test labels withheld)
	Cardiac Acquisition for Multi-structure Ultrasound Segmentation (CAMUS) [62]	2D Echocardiography	LV endocardium, LV myocardium and left atrium	Public registered (test labels withheld)
Breast	Breast Ultrasound Images Dataset(BUSI) [63]	Ultrasound	Breast mass	Public
Liver	Liver Tumor Segmentation Challenge (LiTS) [64]	CT	Liver and liver-tumor segmentation	Public registered (test labels withheld)
Colon	Kvasir-SEG: Kvasir Polyp Segmentation Dataset [65]	Endoscopy	Polyp	Public
Skin	International Skin Imaging Collaboration Challenge Series (ISIC) [66]	Dermoscopy	Skin-lesion segmentation and classification (year-dependent tasks)	Public registered (test labels withheld)
Kidney	Kidney Tumor Segmentation Challenge (KiTS series) [67]	CT	Kidney and kidney-tumor segmentation	Public registered (test labels withheld)
Fetal Head	Head Circumference Ultrasound Dataset (HC) [68]	Ultrasound	Fetal head-circumference plane segmentation	Public registered (test labels withheld)
Abdomen	Fast and Low-resource Abdominal Organ Segmentation Challenge (FLARE series) [69]	CT	Multi-organ segmentation (e.g., liver, spleen, pancreas, kidneys)	Public registered (test labels withheld)
	Whole Organ CT Abdomen Dataset (WORD) [70]	CT	Whole-abdomen multi-organ segmentation (≈16 organs)	Public by request
	Abdominal Multi-Organ Segmentation Challenge (AMOS) [71]	MRI, CT	15-organ abdominal segmentation	Public
	Combined (CT-MR) Healthy Abdominal Organ Segmentation Challenge (CHAOS) [72]	MRI, CT	Liver, spleen, left and right kidney multi-organ segmentation	Public registered (test labels withheld)
Multi-organ	International Symposium on Biomedical Imaging Challenge Series (ISBI) [73]	Multimodal	Mixed challenges across organs	Public registered
	CVPR 2024 MedSAM on Laptop Challenge [42]	Multimodal	Promptable segmentation benchmark across multiple modalities and anatomies	Public registered
	Medical Segmentation Decathlon (MSD) [74]	MRI, CT	10-task benchmark: liver, brain tumor, hippocampus, lung, prostate, heart, pancreas, colon, hepatic vessels, spleen	Public registered (test labels withheld)
Whole body(>100 structures)	TotalSegmentator Dataset [75]	CT	Whole-body segmentation of > 100 anatomical structures (organs, vessels, bones, muscles)	Public

The included datasets span more than twenty organ systems and ten imaging modalities, ranging from widely used public benchmarks such as the Brain Tumor Segmentation (BraTS) dataset, Liver Tumor Segmentation (LiTS) dataset, and Fast and Low-resource Abdominal oRgan sEgmentation (FLARE) challenge dataset, to domain-specific or request-based datasets such as the Whole-body Organ Reconstruction Dataset (WORD) and the Annotated Retinal OCT Images Database for Joint Retinal-Layer and Fluid Segmentation (AROI) dataset. Most datasets are publicly registered with withheld test labels, ensuring fair benchmarking and standardized evaluation across studies.

By systematically organizing these datasets by organ category, modality, clinical focus, and accessibility, this table provides a unified reference framework for future MedSAM-based research, facilitating transparent comparison, methodological reproducibility, and continued progress in the medical foundation model community.

## Model evaluation

### Overview

To provide an overview of evaluation-oriented studies included in this review, Table 4 summarizes representative papers that assessed MedSAM’s generalization, robustness, fairness, and deployability. Each paper lists authorship information, datasets and modalities used, models under comparison, quantitative results, and key findings. This table serves as a roadmap for subsequent subsections, which analyze performance, generalization, prompt sensitivity, fairness, and deployment aspects in detail.

**Table 4:** Summary of evaluation-oriented studies on MedSAM. Note: NR = not reported; qual-only = qualitative results only; fig-only = results shown in figures

Author	Year	Country/Region	Dataset	Image Modality	Models Evaluated	Dice Score	Key Findings
Li [76]	2025	China	In-house 3T CMR of 701 healthy volunteers	Cardiac MRI	SAM, MedSAM	SAM 89.0% MedSAM 86.0%	Shows anatomy-specific fairness disparities across gender, age, and BMI between SAM and MedSAM in cardiac MRI.
Li [77]	2024	China	In‑house 1056‑subject cohort	CT, MRI	SAM, MedSAM, SAT, nnU-Net	SAM 86.7% MedSAM 80.4% SAT 74.9% nnU-Net 93.6%	Shows the fairness disparities of large segmentation foundation models across gender, age, and BMI.
Nguyen [78]	2025	US	589-case public pelvic MRI set	MRI	MedSAM, LiteMedSAM, nnU-Net	MedSAM 64.6% LiteMedSAM 58.9% nnU-Net 88.0%	Evaluates the “out-of-the-box” performance of MedSAM and other segmentation models for pelvic MRI
Nouman [79]	2024	Japan	CHAOS, Kvasir-SEG, DRIVE, BUSI	CT, MRI, Ultrasound, Endoscopy, Fundus	MedSAM	CHAOS: 89.0% Kvasir-SEG: 73.0% BUSI: 76.0% DRIVE: NR	Evaluates MedSAM before and after fine-tuning with both precise and jittered bounding boxes
Kulyabin [80]	2024	Germany	OIMHS, AROI	OCT	SAM 2, MedSAM 2, U-Net	MedSAM 2 OIMHS: 91.3% AROI: 90.9% U-Net OIMHS: 87.1% AROI: 87.9% SAM 2 OIMHS: 35.2% AROI: 20.1%	Compares the point- vs. box-prompt performance of MedSAM 2 and SAM 2
Abaid [81]	2024	Ireland	ImageTBAD	CT	SAM, MedSAM, UniverSeg, UNet	MedSAM 3.9% UniverSeg 25.1% UNet 52.7% SAM Qual-only	Compares the out-of-the-box performance of MedSAM, SAM, UniverSeg and Unet
Nouman [82]	2025	Japan	CHAOS, Kvasir-SEG, DRIVE, BUSI	MRI, CT, Ultrasound, Endoscopy, Fundus	MedSAM (zero-shot and fine-tuned) with prompt types: SBB, RBB, CP, SHA	NR	Examines MedSAM across five modalities under diverse prompt designs and accuracy conditions
Gupta [83]	2025	India	In-house, multicenter Gallbladder Cancer cohort	CT	MedSAM SAM-Adapter TransUNet SAM-Med3D nnU-Net	MedSAM 77.6% SAM-Adapter 56.9% TransUNet 47.9% SAM-Med3D 42.3% nnU-Net 40.8%	Trains and validates 2D and 3D segmentation approaches for GBC on multicenter CT
Marinov [84]	2025	Germany	CVPR24 MedSAM on Laptop challenge	Multiple (11 modalities)	MedSAM and classical pipelines	85.7%	Revisits classical, modality-specific approaches to create efficient and accurate segmentation solutions
Ali [85]	2025	China	Pregnant pelvis MRI dataset, ABUS tumor dataset	MRI, Ultrasound	SAM, MedSAM, SAM-Med2D, U-Net, nnU-Net	SAM 62.4% MedSAM 71.6% SAM-Med2D 76.2% U-Net 78.0% nnU-Net 80.5%	Provides a consolidated view of SAM-based medical segmentation about surveying adaptations and synthesizing open challenges
Casali [86]	2025	Italy	76-patient thigh MRI	MRI	SAM,MedSAM, nnU-Net	SAM 92.5% MedSAM: 92.4% nnU-Net: 92.5%	Compares the performance of SAM and MedSAM against nnU-Net on NMD MRI
Rodrigues [87]	2024	Brazil	MICCAI 2016/2017/2018, ISBI-2015, 3D-MR-MS	MRI	MedSAM	MICCAI 2016: 57.0% MICCAI 2017: 54.0% MICCAI 2018: 69.0% ISBI-2015: 63.0%	Evaluates MedSAM’s generalization for WMH across centers, sequences, orientations and prompt perturbations
Saha [88]	2024	Canada	Retrospective CE-MRI cohort of high-risk HCC patients	MRI	SAM, MedSAM	MedSAM 68.0% SAM 60.0%	Identifies key factors that modulate SAM and MedSAM liver-lesion segmentation on CE-MRI

### Evaluation findings

#### Performance comparison

Several comparative studies, including Ali [85], Nouman [79], Casali [86], and Nguyen [78], evaluated how MedSAM performs relative to SAM and traditional segmentation networks. Results consistently show that MedSAM outperforms the general-purpose SAM by 5–20% in Dice scores on structured modalities such as CT and MRI. However, compared with task-specific networks such as nnU-Net [89] or TransUNet [90], MedSAM still lags in segmenting delicate structures and low-contrast lesions.

Studies also demonstrated that lightweight fine-tuning or modality-specific adaptation can close this gap, enabling MedSAM to match or exceed specialized models on some organ-level tasks. For example, Marinov [84] observed that traditional methods such as Graph Cut and GrabCut can achieve performance comparable to that of foundation models when segmenting structures with simple boundaries, suggesting that model complexity alone does not guarantee superior segmentation quality.

#### Generalization ability

MedSAM’s generalization ability has been extensively tested across diverse modalities and disease contexts. Studies such as Rodrigues [87] and Casali [86] demonstrated that MedSAM shows strong zero-shot generalization on standard CT and MRI datasets but significant degradation when transferred to OCT, ultrasound, or histopathology images. Kulyabin [80] specifically highlighted that SAM-based models, including MedSAM, struggle to capture delicate retinal layers and low-contrast pathological boundaries, with Dice dropping below 0.7 without fine-tuning. Similarly, Nguyen [78] emphasized that domain-specific pretraining remains crucial for specialized anatomical regions. These findings collectively suggest that MedSAM’s performance is sensitive to modality and remains constrained by domain shift between modalities.

#### Prompt sensitivity

Studies such as Nouman [82] and Saha [88] analyzed the influence of different prompt types and input resolutions. Results indicate that prompt configurations, including points and bounding boxes, can cause Dice variation of 5 to 15%. Misplaced or undersized bounding boxes notably reduce boundary precision, whereas higher input resolution improves segmentation of small or thin structures. These experiments highlight that prompt quality and image scale are critical determinants of MedSAM’s accuracy and stability, underscoring the need for automated or adaptive prompting in clinical deployment.

#### Fairness and robustness

Fairness and robustness have become indispensable considerations in evaluating foundation models for medical segmentation. Although MedSAM has demonstrated promising generalization, recent studies revealed that its segmentation performance is not uniformly distributed across demographic groups or imaging conditions, raising concerns about bias and clinical reliability.

Li [76] conducted a comprehensive assessment of MedSAM on cardiac MRI segmentation, involving 701 healthy volunteers stratified by gender, age, and BMI. The study found that MedSAM exhibited substantial fairness gaps across demographic attributes, with up to 10% differences in Dice scores between male and female participants, particularly in vessel segmentation. Moreover, spatial error maps showed distinct sub-regional biases: segmentation errors in males concentrated in the aortic arch and right-ventricular regions, while females exhibited greater errors in peripheral vessels. These disparities suggest that foundation models may implicitly encode population- and anatomy-specific biases, even when trained on large, generalized datasets.

Expanding beyond cardiology, Li [77] also conducted a large-scale fairness analysis on multi-organ MRI and CT datasets covering 1,056 subjects, examining five representative organs: liver, kidney, spleen, lung, and aorta. The results demonstrated that MedSAM showed statistically significant performance disparities across gender, age, and BMI. For example, in abdominal MRI, MedSAM displayed bias in kidney and spleen segmentation.

Collectively, these findings underscore that fairness and robustness are deeply interlinked: demographic bias may arise from insufficient domain adaptation and scanner heterogeneity, while robustness failures across imaging modalities can amplify inequities in clinical outcomes. To achieve equitable deployment, future MedSAM-based systems should integrate fairness-aware loss design, demographically balanced training, multi-center data validation, and standardized fairness benchmarks.

### System framework and workflow

**Fig. 4:**
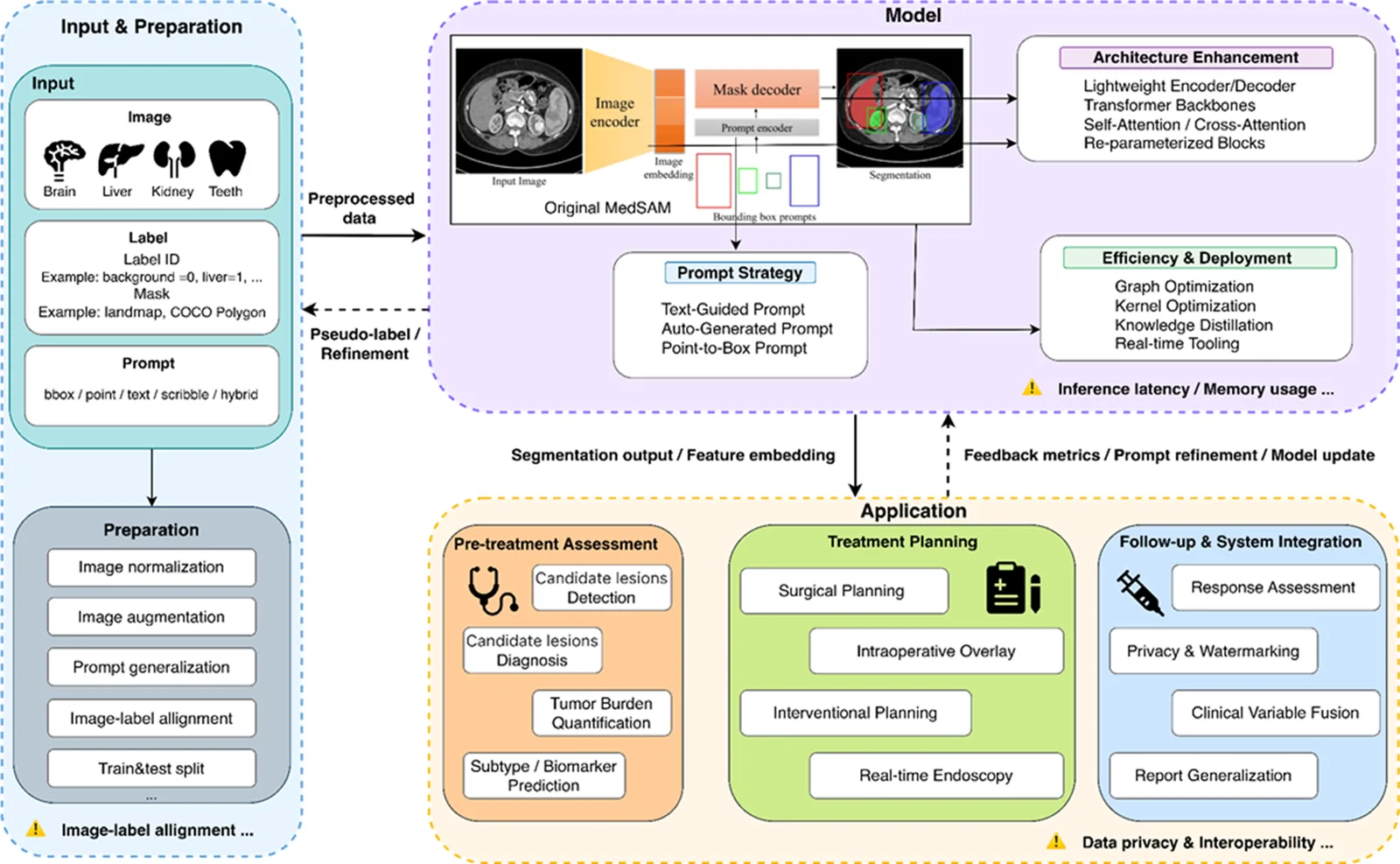
System-level workflow of MedSAM and its derivatives

To provide a holistic understanding of the MedSAM ecosystem, Fig. 4 illustrates the complete workflow encompassing data input, model processing, and clinical application. Rather than functioning as isolated modules, each component in the framework interacts dynamically through continuous data flow and iterative feedback loops. The framework illustrates end-to-end data flow across input, model, and application stages. Arrows indicate both forward information propagation from image preprocessing and prompt generation to segmentation and clinical application, and backward feedback, where model outputs are reintegrated into data refinement and prompt generation for continual improvement. Yellow warning icons mark key bottlenecks, including image-label alignment, inference latency, and data privacy and interoperability, which remain significant challenges for clinical deployment.

At the Input and Preparation stage, raw medical images from various modalities such as CT and MRI are first normalized, augmented, and registered to ensure cross-domain consistency. Prompt generation—including bounding boxes, points, or text—is aligned with corresponding label maps to form the structured input triplet. These preprocessed elements are passed into the Model stage, where the image encoder extracts feature embeddings, the prompt encoder integrates spatial or textual cues, and the mask decoder outputs segmentation maps.

Within the Model stage of the MedSAM workflow, four innovation modules can be identified that collectively define how methodological advances have shaped the MedSAM ecosystem. These modules—Architecture Enhancement, Prompt Strategy, Efficiency and Deployability, and Pipeline and Application—reflect the key directions along which MedSAM has evolved and together constitute the structural foundation of this review. Architecture Enhancement focuses on improving encoder and decoder design to enhance feature representation learning and spatial accuracy through lightweight backbones, hierarchical attention, and reparameterized decoders, reducing computational cost while maintaining anatomical precision. Prompt Strategy governs how spatial, textual, or multimodal prompts guide segmentation, illustrating the transition from purely manual guidance to intelligent, context-aware prompting mechanisms that enhance adaptability and reduce user dependency. Efficiency and Deployability aims to bridge algorithmic excellence and clinical practicality by introducing model compression, quantization, and knowledge distillation to achieve real-time, resource-efficient inference. Finally, Pipeline and Application extends MedSAM beyond segmentation by embedding it into diagnostic and clinical workflows, enabling lesion assessment, surgical planning, and follow-up monitoring in real-world healthcare environments. Together, these four components delineate how innovations at the model stage enable seamless information flow from input preparation to downstream clinical applications, transforming MedSAM from a standalone segmentation model into a comprehensive and adaptable system within the broader medical imaging workflow.

Despite this integrated design, several bottlenecks persist. First, aligning label masks with prompt inputs remains a significant challenge, often limiting cross-dataset reproducibility. Second, inference-time latency and memory usage constrain real-time performance on edge devices. Finally, interoperability and privacy concerns hinder seamless integration into hospital information systems. Addressing these issues through adaptive prompt calibration, efficient model compression, and secure deployment protocols will be essential for advancing MedSAM toward clinical reliability.

## Discussion

To our knowledge, this scoping review provides one of the first multi-dimensional critical appraisals of MedSAM as an emerging foundation-model paradigm for medical imaging. Rather than only cataloging incremental refinements, our synthesis integrates evidence from 45 studies to examine the conditions under which MedSAM could be more broadly deployed across multimodal clinical workflows, and to characterize the algorithmic, safety, fairness, and regulatory considerations that currently constrain that deployment. We established a unified taxonomy delineating four methodological axes—Architecture Enhancement, Prompt Strategy, Efficiency and Deployability, and Pipeline and Application—and mapped these innovations explicitly against evaluation evidence covering performance, generalization, prompt sensitivity, demographic fairness, and clinical applicability. This integrated framework allows us to move beyond description toward an evidence-mapping synthesis of readiness, risk, and research priorities; because this is a scoping review without formal quality appraisal or meta-analysis, these observations should be read as hypothesis-generating priorities for future systematic and prospective research rather than as definitive, evidence-graded conclusions.

Our analysis reveals that MedSAM research has evolved along two parallel trajectories. On the innovation front, recent advances demonstrate a clear shift from computationally intensive architectural modifications toward lightweight, prompt-aware, and deployable models. Encoder enhancements prioritize efficiency through knowledge distillation and parameter-efficient fine-tuning, while decoder refinements focus on spatial precision and cross-modality robustness. Prompt strategies have progressed from manual spatial inputs toward intelligent multimodal and automatic prompting mechanisms that reduce clinician burden while maintaining segmentation accuracy. Efficiency-oriented studies increasingly co-optimize architecture, quantization, and runtime design to achieve real-time inference without compromising fidelity. Application-oriented studies demonstrate the technical feasibility of integrating MedSAM into prototype diagnostic pipelines spanning radiology, surgery, and pathology.

On the evaluation front, our synthesis reveals both strengths and persistent limitations. MedSAM consistently outperforms general-purpose SAM by 5–20% on structured modalities such as CT and MRI but remains inferior to task-specific models like nnU-Net for delicate structures and low-contrast lesions. Zero-shot generalization is strong within similar modalities but degrades substantially when transferred to ultrasound, OCT, or histopathology without fine-tuning. Prompt sensitivity analyses reveal 5–15% Dice variation based on prompt quality and image resolution, highlighting the critical need for adaptive prompting mechanisms. Of particular note, the two available fairness assessments in our corpus report demographic disparities of up to 10% in Dice score across gender, age, and BMI groups, raising the possibility that foundation models may encode population-specific biases despite large-scale training. Because this finding currently rests on two single-center cohorts in specific imaging contexts (cardiac MRI and multi-organ CT/MRI), we treat it as an important signal warranting replication in larger, more diverse, multi-center cohorts rather than as an established, generalizable property of MedSAM.

Despite remarkable methodological progress, current evidence indicates that MedSAM remains primarily at the proof-of-concept stage. Most studies are retrospective and single-center, with limited prospective validation, standardized uncertainty quantification, or regulatory evaluation. This represents a limited level of evidence for clinical translation. While MedSAM demonstrates robust performance on well-defined organ boundaries in structured imaging, it struggles with unstructured, noisy, or low-contrast modalities. These findings suggest that although MedSAM provides a promising foundation for promptable segmentation, the current evidence base does not yet support direct clinical deployment without task-specific fine-tuning, multi-center validation, and further fairness-related research. Three categories of consideration emerged from our synthesis as warranting particular emphasis.

First, clinical safety [91]. The absence of prospective, clinician-in-the-loop studies means that failure modes encountered in retrospective benchmarks, including substantial boundary errors on low-contrast lesions, sensitivity to bounding-box placement, and performance degradation on out-of-distribution modalities such as OCT and ultrasound—have not yet been systematically characterized under real clinical conditions. A missed or mis-delineated boundary in radiotherapy planning or tumor staging could carry patient-safety consequences that aggregate Dice-score averages do not fully capture, although this potential consequence has not itself been directly measured in the studies we reviewed. Future work should consider outcome-oriented safety metrics (e.g., false-negative volume rates for lesion detection, inter-rater agreement on segmentation outputs) and adverse-event reporting in multi-center trials before regulatory submission.

Second, algorithmic bias as a potential patient-safety consideration. The fairness studies report statistically significant segmentation disparities across sex, age, and BMI in cardiac MRI and multi-organ CT/MRI cohorts, respectively, with Dice gaps of up to 10% persisting even after large-scale pre-training. We note that the original studies report nominal *p*-values from t-tests (sex) and correlation analyses (age, BMI) without describing correction for multiple comparisons across organs and strata; the statistical significance of individual subgroup comparisons should therefore be interpreted with appropriate caution pending replication with correction for multiplicity. These disparities are plausible candidates for downstream clinical consequence: biased segmentation of the right ventricle or peripheral vessels in female patients, or of abdominal organs in patients with high BMI, could in principle contribute to differential diagnostic accuracy or treatment-planning error, although neither study directly measured downstream diagnostic or treatment outcomes. The mechanism underlying these biases, whether rooted in training-data imbalance, scanner heterogeneity, or anatomical variation not captured in the pre-training corpus, remains unresolved. Addressing bias would likely benefit from demographically balanced training, fairness-aware loss functions, and standardized subgroup reporting (analogous to subgroup reporting in randomized controlled trials), which journals and regulatory bodies could consider encouraging.

Third, real-world implementation hurdles. Our review reveals a structural gap between algorithmic performance and clinical deployability that goes beyond computational efficiency. Only two systems (SAMME and MODS) have demonstrated integration into operational clinical software (3D Slicer and PACS, respectively), and both remain at pilot stage. The barriers are multi-layered: (a) workflow interoperability—MedSAM should interface with DICOM-compliant PACS and RIS systems under time constraints imposed by radiology reporting workflows; (b) regulatory compliance—FDA Software as a Medical Device (SaMD) clearance and EU CE marking require prospective performance evidence that no current MedSAM study provides; (c) data governance—HIPAA and GDPR compliance for federated or multi-center deployments demands privacy-preserving architectures (federated learning, differential privacy) that are nascent in the MedSAM literature; and (d) human-AI trust calibration—clinicians should understand when to override model outputs, necessitating calibrated uncertainty quantification and explainability features absent from most current implementations [92]. These are not secondary engineering concerns; they are among the key determinants of whether MedSAM’s algorithmic advances will translate into patient benefit.

The MedSAM ecosystem offers substantial opportunities to advance foundation models in medical imaging [92]. The emergence of cross-modality and multi-task frameworks enables generalizable medical representations that integrate CT, MRI, ultrasound, and endoscopy within a unified latent space, reducing reliance on modality-specific fine-tuning. Volumetric and temporal variants such as MedSAM2 [93] extend prompting to 3D and video data, paving the way for longitudinal monitoring, motion-aware radiotherapy planning, and intraoperative navigation. Meanwhile, adaptive and context-aware prompting mechanisms, which combine automatic prompt generation with multi-scale attention, offer pathways toward intelligent, clinician-in-the-loop systems that improve usability and robustness. Integrating MedSAM with diagnostic reasoning and generative frameworks could further enable multimodal, multifunctional pipelines that link segmentation, classification, and report generation, thereby bridging perception and decision-making in clinical practice. Finally, the expansion of multi-center datasets and public challenges such as the CVPR MedSAM Challenge [42] provides a foundation for standardized evaluation, reproducible benchmarking, and transparent model validation.

Nevertheless, significant challenges remain before MedSAM can achieve routine clinical translation. Data and domain heterogeneity across institutions, imaging protocols, and populations still cause substantial performance variation, underscoring the need for domain-adaptive and fairness-aware training. From a regulatory perspective, translating MedSAM into clinical workflows requires compliance with pathways such as U.S. FDA clearance [94] and European CE marking for medical AI software [95]. Ethical and governance frameworks, such as the EU AI Act and the Belmont Report principles, are essential to ensure human oversight, transparency, and accountability in prompt-driven decision-making [96]. Furthermore, compliance with the Health Insurance Portability and Accountability Act (HIPAA) [97] in the U.S. and the General Data Protection Regulation (GDPR) [98] in the EU is critical to guarantee data privacy, particularly for cross-institutional and federated learning scenarios. Computational scalability also poses barriers for smaller research centers, while interoperability with existing PACS and radiology reporting systems remains technically challenging [99, 100]. Addressing these issues will require multidisciplinary effort involving researchers, clinicians, ethicists, and regulators to establish trustworthy and equitable frameworks for clinical AI deployment.

Looking forward, we identify several converging research directions critical for advancing MedSAM toward clinical maturity. First, developing unified architectures that integrate segmentation with diagnostic reasoning and report generation will enable multifunctional medical AI systems. Second, extending MedSAM to volumetric and temporal domains (3D and 4D) will unlock applications in longitudinal monitoring, motion-aware radiotherapy, and intraoperative guidance [101]. Third, implementing self-adaptive prompting with uncertainty-aware mechanisms will enhance robustness and reduce reliance on manual intervention. Fourth, establishing standardized, fairness-aware evaluation protocols with multi-center validation will ensure equitable performance across diverse patient populations and imaging conditions. Finally, addressing regulatory compliance, data privacy, and interoperability with existing clinical systems (PACS, RIS) will be essential for real-world adoption.

From a broader perspective, several critical challenges should be addressed before widespread clinical integration. Data and domain heterogeneity across institutions, protocols, and demographics continue to cause substantial performance variation. Regulatory pathways including FDA clearance and CE marking require prospective validation studies demonstrating safety and efficacy. Ethical governance frameworks should ensure human oversight, transparency, and accountability, particularly in prompt-driven decision-making [102]. Privacy compliance with HIPAA and GDPR is essential for cross-institutional collaboration and federated learning [103]. Computational scalability remains a barrier for resource-limited centers, while seamless integration with existing radiology workflows requires substantial engineering effort.

In summary, MedSAM is transitioning from methodological development toward clinical evaluation. Its rapid progress in representation learning, multimodal fusion, and prompt intelligence should be matched by rigorous validation, ethical accountability, and real-world usability to ensure safe, transparent, and sustainable adoption in healthcare [104].

### Limitations

This review has several limitations. First, only English-language studies were included, which may have introduced language bias and excluded valuable contributions from non-English-speaking countries. Second, given the rapid evolution of foundation models, some recent preprints may not have been captured, potentially limiting temporal completeness. Relatedly, a minority of the included studies are preprints that had not completed formal peer review at the time of our search; we retained them, consistent with the rapid dissemination norms of this subfield, but their conclusions should be regarded as provisional until they appear in the peer-reviewed literature. Third, because MedSAM is a relatively new model, the available evidence base is necessarily small and rapidly evolving, and the breadth statistics should therefore be read as indicators of the scope and diversity of the literature rather than as a graded evidentiary basis for the strength of any individual claim. Fourth, quantitative meta-analysis was not performed given heterogeneity in outcome measures, datasets, and task definitions across studies; pooled effect estimates would have been misleading. Future reviews should prioritize standardized reporting (e.g., model cards, fairness scorecards) to enable formal meta-analysis.

## Conclusion

MedSAM marks a notable milestone in the foundation-model era of medical imaging, bridging large-scale pretraining and domain specialization. Our synthesis indicates that the field has made substantial technical progress across its multimodal innovation axes: lightweight architectures enable real-time CPU inference; multimodal and automated prompting mechanisms have the potential to reduce clinician burden; and early integration pipelines spanning radiology, surgery, and pathology illustrate that MedSAM could plausibly function as a modular backbone within clinical imaging workflows, although this remains to be demonstrated at scale. The safety, bias, and implementation gaps documented here do not negate this progress, but they do indicate that the evidence base has not yet matured to a point that would support broad clinical deployment. The limited fairness evidence currently available, drawn from two single-center cohorts, raises a concern about demographic performance variation that merits dedicated, larger-scale investigation before its clinical significance can be established. Absent prospective multi-center validation, clearer regulatory pathways, and workflow interoperability standards, the promise of multimodal MedSAM-based systems will remain largely unrealized in routine practice. Clinical translation will require prospective multicenter validation, transparent model-update procedures, and compliance with applicable medical-device regulations. This review provides a structured map of MedSAM-related innovations, evaluation evidence, and gaps in fairness, safety, and regulatory readiness. We suggest that algorithmic performance be considered alongside the standards of evidence expected of any medical device or software: prospective evaluation, attention to demographic equity, and institutional accountability. The ultimate measure of MedSAM’s impact should be improved patient outcomes across diverse populations, rather than segmentation metrics alone, and we offer this review as a structured starting point for the prospective, fairness-aware research needed to assess that impact.

## Electronic supplementary material

Below is the link to the electronic supplementary material.

Supplementary material 1

## Data Availability

All data extracted for this review are presented in the article and its supplementary materials.
