# Supplementary material for: Medical Segment Anything Model (MedSAM): methodological innovations, evaluation practices, and clinical applications
Source: BMC Med Imaging. 2026 Jul 29;26:544. doi: 10.1186/s12880-026-02600-z (PMC13700131; doi:10.1186/s12880-026-02600-z)
Supplement: Supplementary file 1 [file 12880_2026_2600_MOESM1_ESM.docx]

**Supplemental Tables**

**Supplemental Table 1.** Search strategies for all the databases

| **Database** | **Search String** |
| --- | --- |
| **PubMed/MEDLINE** | ("Diagnostic Imaging"[Mesh] OR diagnostic-imag*[tiab] OR radiolog*[tiab] OR radiograph*[tiab] OR tomograph*[tiab] OR echograph*[tiab] OR ultrasound*[tiab] OR ultrasonograph*[tiab] OR sonogram*[tiab] OR Fluoroscopy*[tiab] OR fluorescen*[tiab] OR radiofluoroscop*[tiab] OR Radiodiagnos*[tiab] OR diagnostic-radiolog*[tiab] OR radiologic-diagnos*[tiab] OR radioimag*[tiab] OR magnetic-resonance*[tiab] OR MRI[tiab] OR x-ray*[tiab] OR Echocardiograph*[tiab] OR ECG[tiab] OR EKG[tiab] OR medical-imag*[tiab] OR CT-imag*[tiab]) AND (segment-anything-model*[tiab] OR medSAM[tiab] OR "medical SAM"[tiab] OR "Medical Segment Anything Model"[tiab]) |
| **Embase** | ('Diagnostic Imaging'/exp OR diagnostic-imag*:ti,ab OR radiolog*:ti,ab OR radiograph*:ti,ab OR tomograph*:ti,ab OR echograph*:ti,ab OR ultrasound*:ti,ab OR ultrasonograph*:ti,ab OR sonogram*:ti,ab OR 'Fluoroscopy'/exp OR fluorescen*:ti,ab OR radiofluoroscop*:ti,ab OR Radiodiagnos*:ti,ab OR diagnostic-radiolog*:ti,ab OR radiologic-diagnos*:ti,ab OR radioimag*:ti,ab OR 'Magnetic Resonance Imaging'/exp OR MRI:ti,ab OR x-ray*:ti,ab OR 'Echocardiograph'/exp OR ECG:ti,ab OR EKG:ti,ab OR medical-imag*:ti,ab OR CT-imag*:ti,ab) AND (segment-anything-model*:ti,ab OR medSAM:ti,ab OR "medical SAM":ti,ab OR "Medical Segment Anything Model":ti,ab) |
| **Web of Science** | TS=("Diagnostic Imaging" OR diagnostic-imag* OR radiolog* OR radiograph* OR tomograph* OR echograph* OR ultrasound* OR ultrasonograph* OR sonogram* OR Fluoroscopy OR fluorescen* OR radiofluoroscop* OR Radiodiagnos* OR diagnostic-radiolog* OR radiologic-diagnos* OR radioimag* OR magnetic-resonance* OR MRI OR "x-ray*" OR Echocardiograph* OR ECG OR EKG OR medical-imag* OR CT-imag*) AND TS=("segment-anything-model*" OR medSAM OR "medical SAM" OR "Medical Segment Anything Model") |
| **Scopus** | TITLE-ABS-KEY("Diagnostic Imaging" OR diagnostic-imag* OR radiolog* OR radiograph* OR tomograph* OR echograph* OR ultrasound* OR ultrasonograph* OR sonogram* OR Fluoroscopy OR fluorescen* OR radiofluoroscop* OR Radiodiagnos* OR diagnostic-radiolog* OR radiologic-diagnos* OR radioimag* OR "magnetic-resonance*" OR MRI OR "x-ray*" OR Echocardiograph* OR ECG OR EKG OR medical-imag* OR CT-imag*) AND TITLE-ABS-KEY("segment-anything-model*" OR medSAM OR "medical SAM" OR "Medical Segment Anything Model") |
| **IEEE Xplore** | ("All Metadata":("diagnostic imag*" OR radiolog* OR radiograph* OR tomograph*  OR echograph* OR ultrasound* OR ultrasonograph* OR sonogram* OR fluoroscopy*  OR fluorescen* OR radiofluoroscop* OR radiodiagnos* OR diagnostic-radiolog*  OR radiologic-diagnos* OR radioimag* OR magnetic-resonance* OR MRI OR x-ray*  OR echocardiograph* OR ECG OR EKG OR medical-imag* OR CT-imag*))  AND  ("All Metadata":("segment anything model*" OR medSAM OR "medical SAM"  OR "Medical Segment Anything Model")) |
| **ACM Digital Library** | AllField:(  "diagnostic imag*" OR radiolog* OR radiograph* OR tomograph* OR echograph*  OR ultrasound* OR ultrasonograph* OR sonogram* OR fluoroscopy* OR fluorescen*  OR radiofluoroscop* OR radiodiagnos* OR diagnostic-radiolog* OR radiologic-diagnos*  OR radioimag* OR magnetic-resonance* OR MRI OR x-ray* OR echocardiograph*  OR ECG OR EKG OR medical-imag* OR CT-imag*  )  AND  AllField:(  "segment anything model*" OR medSAM OR "medical SAM" OR "Medical Segment Anything Model"  ) |
